# Label-Free Biosensor Imaging on Photonic Crystal Surfaces

**DOI:** 10.3390/s150921613

**Published:** 2015-08-28

**Authors:** Yue Zhuo, Brian T. Cunningham

**Affiliations:** 1 Department of Bioengineering, University of Illinois at Urbana-Champaign, Champaign, IL 61822, USA; E-Mail: yuezhuo2@illinois.edu; 2 Department of Electrical and Computer Engineering, University of Illinois at Urbana-Champaign, Champaign, IL 61822, USA

**Keywords:** photonic crystal, photonic crystal surface, photonic crystal biosensor, photonic crystal enhanced microscopy (PCEM), photonic crystal enhanced fluorescence (PCEF), label-free bioimaging, nanophotonics, biomaterial detection, live cell imaging, nanoparticle detection, protein-protein binding detection

## Abstract

We review the development and application of nanostructured photonic crystal surfaces and a hyperspectral reflectance imaging detection instrument which, when used together, represent a new form of optical microscopy that enables label-free, quantitative, and kinetic monitoring of biomaterial interaction with substrate surfaces. Photonic Crystal Enhanced Microscopy (PCEM) has been used to detect broad classes of materials which include dielectric nanoparticles, metal plasmonic nanoparticles, biomolecular layers, and live cells. Because PCEM does not require cytotoxic stains or photobleachable fluorescent dyes, it is especially useful for monitoring the long-term interactions of cells with extracellular matrix surfaces. PCEM is only sensitive to the attachment of cell components within ~200 nm of the photonic crystal surface, which may correspond to the region of most interest for adhesion processes that involve stem cell differentiation, chemotaxis, and metastasis. PCEM has also demonstrated sufficient sensitivity for sensing nanoparticle contrast agents that are roughly the same size as protein molecules, which may enable applications in “digital” diagnostics with single molecule sensing resolution. We will review PCEM’s development history, operating principles, nanostructure design, and imaging modalities that enable tracking of optical scatterers, emitters, absorbers, and centers of dielectric permittivity.

## 1. Introduction

**Figure 1:**
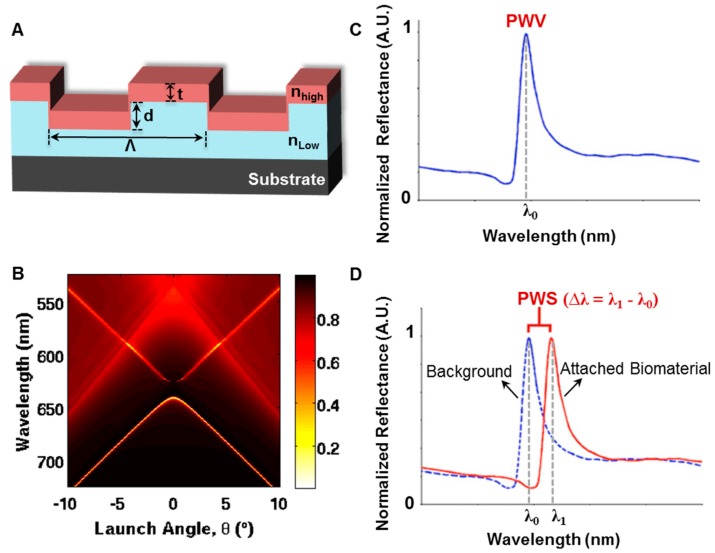
Photonic Crystal (PC) Surface Biosensor. (**A**) Schematic of the PC surface on a substrate with structure parameters: grating period (Λ), grating depth (d), refractive index (RI) of low-RI grating material (n_low_) and high-RI top layer (n_high_), thickness of high-RI layer (t); (**B**) Band structure of a photonic crystal biosensor calculated by FDTD simulation; (**C**) Normalized reflection spectrum from the PC surface with resonant peak wavelength value (PWV) of λ_0_; (**D**) Peak wavelength shift (PWS) of Δλ extracted from the normalized spectra with a background pixel (PWV of λ_0_) and a pixel with surface-attached biomaterial (PWV of λ_1_) on the PC biosensor.

**Figure 2:**
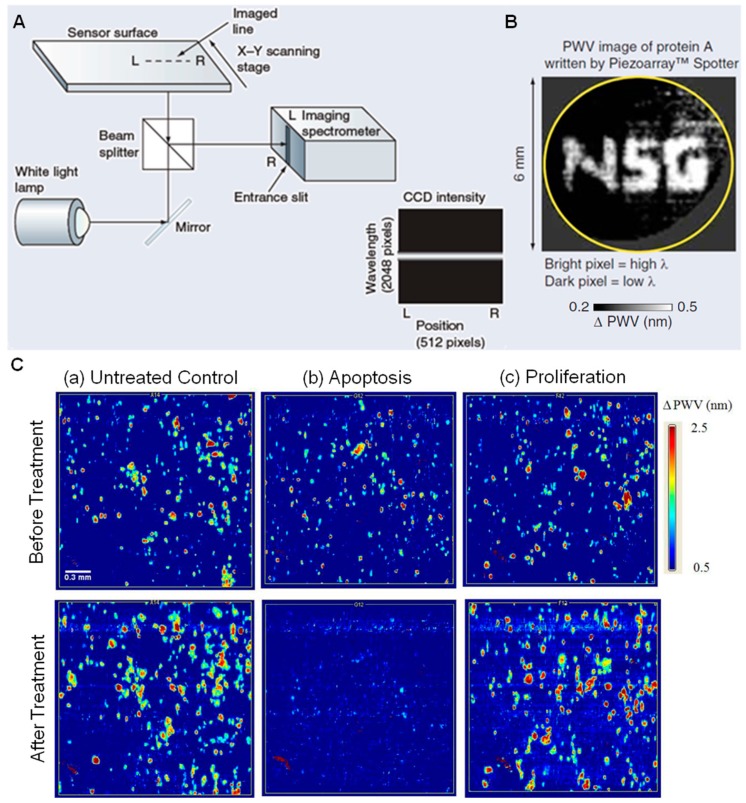
Instrument 1: Label-free Biomolecular Interaction Detection (BIND) Scanner utilizing a PC surface biosensor. (**A**) Schematic of excitation/detection instrument where an imaging spectrometer gathers hundreds of reflected spectra simultaneously from one line across the sensor surface; (**B**) PWS images of Protein A (bright regions represent regions of greater PWS) gathered on a 6-mm diameter region of a PC biosensor, which is imaged at approximately 20 µm pixel resolution after writing the letters ‘NSG’ (Nano Sensors Group) with a microarray spotting tool (PerkinElmer, Inc. Piezoarray™) (Reprinted in part with permission from [50], © 2006 Future Drugs Ltd.); (**C**) PWS images with shift scale bars (ΔPWV) indicating the magnitude of wavelength shifts in nanometers. Pixels with higher PWS displayed in brighter colors indicate locations where Panc-1 cell attachment has occurred. The three columns of image sets represent the following: (a) untreated control, (b) extract that induced 100% cell death Petunia punctata Paxton (*P. punctate*), (c) extract that enhanced proliferation Anisoptera glabra Kurz (*A. glabra*). The top row of images was taken before exposure and the bottom row of images was taken after the 24 h exposure period with a plant extract at 100 μg/mL. Scale bar (white) = 300 μm (Reprinted in part with permission from [41], © 2010 BioMed Central Ltd.).

**Figure 3:**
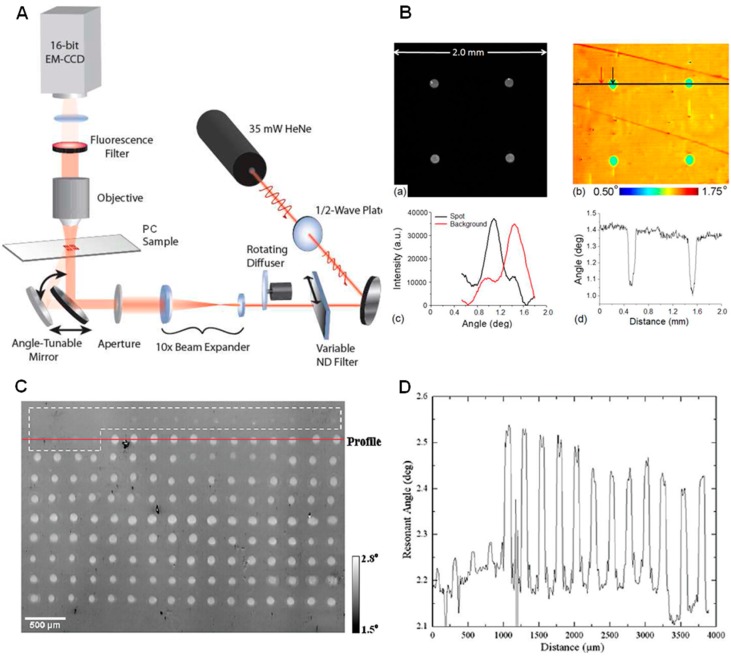
Instrument 2: Transmission acquisition mode of photonic crystal biosensor integrated with an upright imaging microscope and using laser as light source. (**A**) Schematic of combined label-free and enhanced-fluorescence imaging instrument; (**B**) Enhanced (a) fluorescence and (b) label-free images of 50 mg/mL SA-Cy5 spots on a PC biosensor. Inverted transmission *versus* angle response for a pixel inside and outside the SA-Cy5 spot in (c), and cross-section of the label-free image through two SA-Cy5 spots in (d). Rather than measuring the PWS, the label-free imaging system measures the angle of minimum transmission (AMT) by illuminating the PC sensor at a fixed wavelength while scanning the angle of illumination through computer-controlled rotation of the mirror (reprinted in part with permission from [76], © 2009 American Optical Society); (**C**) Label-free image of a DNA microarray measured with a PC biosensor. The white dashed box denotes the location of a set of 20 intentional blank spots. A line profile running through a row containing 4 blank spots followed by 12 probe spots is shown in (**D**) (Reprinted in part with permission from [37], © 2010 American Chemical Society).

**Figure 4:**
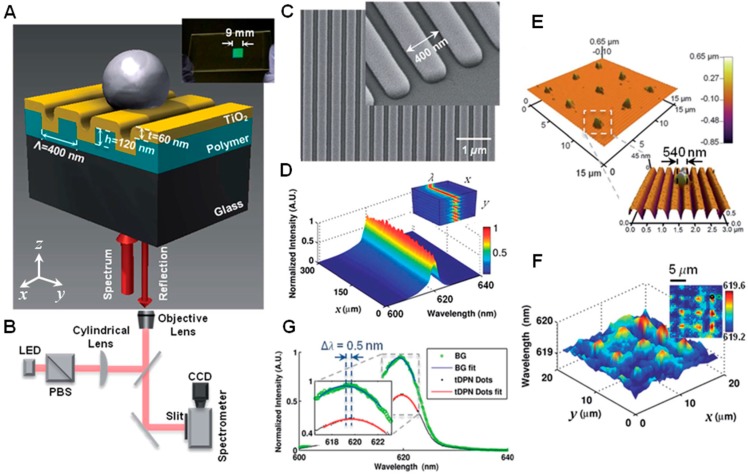
Instrument 3: Reflection acquisition mode of photonic crystal biosensor integrated with an inverted microscope and using LED as light source. (**A**) Schematic of the structure of a photonic crystal (PC) surface biosensor with a surface-attached nanoparticle, inset: photo of a PC biosensor fabricated on a glass slide; (**B**) Instrument schematic of the modern Photonic Crystal Enhanced Microscopy (PCEM); (**C**) Scanning electron micrograph of the photonic crystal surface, inset: zoomed-in image on the edge of the PC biosensor; (**D**) Normalized spectrograph (surface plot) measured with PCEM. Inset: PCEM-acquired 3D spectrum data; (**E**) AFM images of a tDPN-printed 3 × 3 array of nano-dots (each with dimension of 540^2^ × 40 nm^3^), inset: zoomed-in AFM image of one tDPN-printed dot; (**F**) PWV image of the tDPN-printed dots (displayed in a 3D surface plot) within a 20^2^ µm^2^ field of view, inset: 2D PWV image; (**G**) Normalized spectra of a representative tDPN-printed dot and a background pixel, inset: zoomed-in image of the spectra with 2D polynomial fitting (Reprinted in part with permission from [48], © 2014 RSC Publishing.).

A photonic crystal (PC) surface is a periodic-modulated dielectric nano-structure material (one example can be seen in Figure 1A) [1,2,3,4,5]. PC surfaces can be designed to provide photonic bandgaps (Figure 1B), within which light propagation is prohibited for specific wavelengths [6,7,8]. Therefore, the local optical modes provided by the PC surface can be utilized as a highly sensitive and label-free platform for biosensing and bioimaging in life science research. PC surface biosensors [9,10,11,12,13,14,15,16,17,18,19,20,21,22,23,24,25,26,27,28,29,30] have been widely used to detect refractive index changes induced by surface-attached biomaterials (Figure 1C,D), and for analytes spanning a wide range of dimensions, including small molecules [31,32,33,34,35], virus particles [36], DNA microarrays [37], and live cells [38,39,40,41,42,43,44,45]. Generally, biosensing is realized with a transducer surface (e.g., PC surface, waveguide or microcavity) and an instrument for collecting the average response from the entire sensing area. When spatially resolved information is required, such as the behavior within individual cells, it is necessary to measure localized responses that can be differentiated from neighboring locations. Thus, spatial resolution becomes a critical factor for biosensor imaging. Among the earliest developed label-free imaging modalities based on PC biosensors [12,16,38,46,47], Photonic Crystal Enhanced Microscopy (PCEM) [12,38,44,45,46,47,48,49,50,51] represents a new form of optical microscopy that uses a PC surface to dynamically detect and visualize biomaterial-surface interactions (Figure 2, Figure 3 and Figure 4). Because the detection is label-free, it is not limited by the transient activity of fluorescent contrast agents that may be limited by photobleaching effects. Hence, PCEM can be performed for extended time periods to enable study of cell functions (including cell adhesion, migration, apoptosis, and differentiation) that take place over the course of several hours or multiple days.

Based on the number of directions with a periodic repetition of refractive index (RI) contrast, PC nano-structures can be categorized as one-dimensional (1D), two-dimensional (2D), or three-dimensional (3D). A PC surface typically consists of an area of continuous 1D or 2D PC structure on the substrate surface. Here we describe the case of a 1D PC structure as an example to explain label-free biosensor imaging on PC surfaces. Traditionally, a 1D PC is characterized as a multilayer stack of materials with alternating dielectric constants, which are also referred to as Bragg mirrors (or dielectric mirrors) [52,53,54,55,56,57,58,59]. In such a 1D PC stack, the periodicity is normal to the substrate plane and a photonic bandgap is formed for light with the evanescent part of the wavevector (which is highly sensitive to surface RI modifications) normal to the substrate surface. When used in biosensing and bioimaging, this PC structure utilizes the surface electromagnetic waves bound to the multilayer (named Bloch surface waves or surface electromagnetic waves) to measure the dielectric changes at the substrate surface. However, this type of PC structure has not been used for realizing high spatial resolution biosensor imaging since its Bloch surface modes are not confined laterally (rather they propagate along the plane of the substrate surface). Another type of important PC structure is the PC slab, which consists of a periodicity of RI contrast in the plane of the substrate surface introduced by alternating a high-RI guiding layer (e.g., TiO_2_, GaAs) with low-RI materials (e.g., air, water, Si) [7,27,60,61,62,63,64,65,66,67,68,69,70,71,72,73,74]. The PC slabs are typically comprised of 1D (e.g., linear) or 2D (e.g., quadratic and triangular) structures [7,46,51,63,75], and here we focus on the 1D PC slab since it is the simplest to use for PCEM. A PC slab not only supports in-plane guided modes that are confined by the slab completely (which cannot couple to external radiation), but also supports guided-mode resonances (referred to as quasi guided modes or leaky modes) which can couple to the external environment. Therefore, the maximum intensity of the electromagnetic field can be observed both in the high RI layer and in the evanescent part outside of the PC slab. When used in biosensing and bioimaging, the binding events of biomaterials interacting with the evanescent field atop of the PC slab and the associated RI changes can be obtained by detecting the peak wavelength shift (PWS) of guided-mode resonances in the reflection/transmission spectrum. Since the periodicity is in the plane, the lateral propagation of the modes is prohibited in the PC slab biosensor and, therefore, high spatial resolution can be realized in bioimaging.

The advantages of PCEM are inherent from the optical properties of slab-based PC surfaces, since they can be designed as a wavelength-selective optical resonator and functionalized as a sensitive optical transducer. For instance, high spatial resolution (in-plane) can be achieved in bioimaging due to the restricted lateral propagation of electromagnetic waves on surface of the PC slab. Enhanced electromagnetic fields (in the form of an evanescent field) near the PC surface (penetration depth of ~200 nm) only illuminate surface-adsorbed biomaterials, such as the extracellular matrix (ECM), membrane components of surface-adsorbed cells, or cellular surface-attached nanoparticle tags. This near-field high-intensity illumination regime promises a high axial resolution (out-of-plane) of ~200 nm, which is beyond the diffraction limit in the spectrum-range of the visible light (400–700 nm) during bioimaging. Compared to the broadband resonances and lossy modes (due to absorption) on metal surfaces, narrow line width (e.g., a few nm) resonant spectra and high reflection efficiency (e.g., nearly 100%) on dielectric surfaces of PC biosensors enable measurement of resonant wavelength shifts with high spectral resolution. The PC resonant mode can be measured in a noncontact detection instrument configuration, in which normal incident-angle illumination results in simple integration with a standard microscope. The resonant wavelength can be selected on a PC surface by tuning its geometry (e.g., grating period) or the incident angle of illumination. Thus, the sensing and imaging can be realized in many spectral ranges, including ultraviolet, visible, and infrared (IR). Although PC surfaces have been fabricated by expensive and time-consuming approaches (such as electron-beam (e-beam) or nano-imprint lithography), recent developments in high-throughput and large-area polymer-based techniques (such as nanoreplica molding at room temperature) have led to the commercial introduction of single-use disposable PC sensors that can be manufactured in a roll-to-roll fashion. These PC sensors can be subsequently integrated with standard format microplates, microscope slides, and microfluidic devices for high-throughput drug or cytotoxicity screening of biomolecule or cell assays. The goal of this review is to summarize the genesis, development, and recent advances of PCEM.

## 2. Principles of Modern PCEM

### 2.1. Photonic Crystal Surface Biosensor

A dielectric PC surface (linear PC slab) is utilized as the optical transducer for RI sensing in the label-free PCEM imaging system, as shown in Figure 4A. The PC surface is a resonant grating structure with periodic modulation of the dielectric permittivity of a low-RI material in one dimension (1D) (which provides the nano-pattern) and is then coated with a thin layer of high-RI material (which supports the guided-mode resonances) [49,51]. When illuminated with broadband polarized light, the incident light is coupled into the resonant modes of the PC if the Bragg condition is satisfied. As mentioned earlier, such guided-mode resonances are referred to as “quasi guided modes” or “leaky modes” since they are not allowed to propagate laterally (due to fact that these modes are rapidly re-radiated out from the grating structure) and, thus, have a finite lifetime in the PC structure. Therefore, the resulting electromagnetic standing waves that occur at the resonant wavelength inhibit lateral propagation and open the potential for the PC surface to be utilized for label-free bioimaging. At the combination of incident angle and incident wavelength that satisfies the resonant coupling condition, nearly no light is transmitted through the PC and a high reflection coefficient (~100%) can be achieved during bioimaging [1,2,3]. The input light can be coupled into the PC resonant mode via wavelength or angle control, which does not require high precision position control and, thus, reduces the complexity of the overall imaging instrument.

Fabrication of the PC surface can be performed upon large-area plastic sheets using a roll-to-roll replica-molding procedure that is performed at room temperature [46,77,78,79]. The molding template, which can be used repeatedly (up to thousands of times), can be made on silicon wafers or quartz substrates by deep-UV lithography, nano-imprint lithography, or e-beam lithography. During the replica-molding procedure, a thin layer of liquid ultraviolet-curable polymer (UVCP) (low-RI) is deposited on the molding template and then compressed against the device substrate to create a negative volume image of the grating structure from the mold. After exposure to high-intensity UV light, the UVCP is cured to a solid-phase grating structure (e.g., grating period of Λ = 400 nm, grating depth of *d* = 120 nm, duty cycle of *f* = 50%). A thin layer of high-RI material (e.g., titanium dioxide (TiO_2_)) is subsequently deposited on top of the low-RI grating structure (UVCP), with its thickness (e.g., thickness of *t* = 80 nm) selected to generate a resonant reflection at a specific wavelength (e.g., resonant wavelength of λ_0_ = 620 nm). A scanning electron microscope (SEM) image of a fabricated 1D PC surface is shown in Figure 4C. This replica-molding method provides a rapid, reliable, and inexpensive manufacturing process for PC surface fabrication.

The main criteria for measuring the performance of a PC surface biosensor include sensitivity and spatial-resolution. The sensitivity of a PC biosensor is determined by the material (e.g., the dielectric property of the high-RI layer) or the geometry of the nano-structure (e.g., the thickness of the high-RI layer) [80]. The sensitivity can be estimated with Finite-difference time-domain (FDTD) computer simulations and experimentally characterized with an optical transmission/reflection setup. As mentioned earlier, the spatial resolution of the PC biosensor can be decomposed into in-plane and axial resolution [81]. The in-plane resolution is characterized by the propagation length of resonant modes along the surface plane of the nano-structure and the axial resolution is determined by the penetration depth of the evanescent field atop of the PC surface. In addition, since the PC surface is an optical biosensor, the selectivity is realized by coating the surface-immobilized antibody or ECM molecules on the top of the biosensor. The absence of selectivity constraints on the biosensor avoids the specific design for each application and, thus, enables a broad range of bio-applications for the PC biosensor.

### 2.2. PCEM Imaging Modality and Operating Principle

As shown in the schematic diagram (Figure 4B), the PCEM detection instrument uses a linear scanning approach and is built upon the body of an inverted microscope (Carl Zeiss Axio Observer Z1). In addition to ordinary brightfield imaging, a second illumination path is provided from a fiber-coupled broadband LED, which is incident on the PC from below. The unpolarized LED output light passes a polarized beam splitter (PBS) to illuminate the PC with light polarized with its axis perpendicular to the grating lines (e.g., *y* direction), representing the transverse magnetic (TM) mode. Since the resonant wavelength of a 1D PC surface is only sensitive to the incident angle in one angular dimension (perpendicular to the grating) (*y* direction), the light can be focused in the orthogonal angular dimension (parallel to the grating) (e.g., *x* direction) to strengthen the incident intensity. Therefore, the light passing through the PBS is focused in one axis (*x* direction) by a cylindrical lens, while the light remains collimated in the other angular dimension (*y* direction). The linear beam (collimated direction) is focused on the back focal plane of the objective lens of the microscope. The light emerging from the objective lens (upwards) is thus incident on the PC, so it is collimated in the direction perpendicular to the PC grating lines (*y* direction) and, thus, all the light reaching the PC with the TM polarization has the same angle of incidence. The reflected light beam passes through the objective lens in the opposite direction (downwards), after which it is projected onto an imaging spectrometer through a narrow entrance slit. The imaging spectrometer contains a diffraction grating that disperses the wavelength components of the PC-reflected light. Once the spectrometer is determined, the dimension of one imaged pixel of the PC in the direction parallel to the grating lines (*x* direction) is determined by the magnification of the objective lens and the dimension of pixels within the charge-coupled device (CCD) camera (Photometrics Cascade, 512^2^ pixels). A motorized stage (Applied Scientific Instruments, MS2000) linearly translates the PC in the perpendicular direction to the grating (*y* direction). The step-size of the stage (together with the magnifications of the objective lens) determines the pixel size of the PCEM imaging system in the *y* direction. Therefore, a large area can be scanned in a line-by-line fashion by translating the PC sensor in steps perpendicular to the linear grating direction (*y* direction). For example, with a 10 × objective lens of the microscope, a 16 µm^2^ pixel size of the CCD camera, and a 0.6 µm step size of the motorized stage, a final acquired image with 0.6^2^ µm^2^ pixel size can be measured in PCEM (with an acquisition speed of ~10 s per frame for a scanning area of 300^2^ µm^2^).

For PCEM data acquisition, the linear light beam reflected from the PC that contains the resonant biosensing signal produces a spatially resolved spectrum for each point along the line with a narrow bandwidth (e.g., Δλ ~ 4 nm) and forms a 2D spectrograph (e.g., 512^2^ pixels) across the line (Figure 4D). After line-by-line scanning, a 3D spectrum data (e.g., 512^3^ cube) can be acquired (Figure 4D inset) and the signal/image processing can be performed with computational software (Matlab, MathWorks). Specifically, the spectrum signal can be mathematically fit with a second-order polynomial or Lorentz function for each pixel to extract the peak wavelength and intensity values. With a background image acquired beforehand, shifts in the peak wavelength value (PWV) or shifts in the local peak intensity value (PIV) can be calculated at each pixel location to measure the redistribution of the attached biomaterials.

## 3. History of PCEM Development

The development of PCEM instrumentation can be described chronologically in three main phases that have led to increasingly finer spatial-resolution and illumination/detection optics, which have been designed for scanning biomolecular layers on dry PCs or cell attachment on PC surfaces exposed to liquid media.

### 3.1. Instrument 1—Biomolecular Interaction Detection (BIND) Scanner

In 2002, the first PC biosensor introduced by SRU company (SRU Biosystems) was designed for high-throughput microplate-based detection of protein-protein and protein-small molecule interactions, using a PC with resonant reflection in the near-infrared (NIR) spectral range [49,51]. Shortly afterwards, a PC biosensor microplate reader was introduced that incorporated a linear array of optical fibers with illumination/detection heads that could read all the wells in one row (e.g., *y* direction) of a 96-well microplate at one time [46]. The illumination/detection heads were installed beneath the microtiter plate, which sits upon a motion stage that could translate the plate in an orthogonal dimension (e.g., *x* direction) to scan the entire microplate in ~15 s. This mode enabled serial re-scanning of the microplate to generate kinetic data for the biomolecular interaction taking place in all the wells. The PC biosensor resonant PWV was determined at each location with this linear scan mode. Subsequently, the first-generation label-free PC biosensor imaging system was introduced and named the “Biomolecular Interaction Detection” (BIND) Scanner (Instrument 1, Figure 2A) [12,47,50]. The optical fiber-based illumination/detection approach was replaced by free space illumination of the bottom surface of the PC biosensor with a broadband light source, and the collection of reflected light into an imaging spectrometer, which was able to rapidly acquire a spatial PWV map by scanning a large sensor surface area. Following the light path of the system, the incoming light beam was divided by a beam splitter, directed to the PC biosensor surface, magnified by an optional objective lens, and, finally, projected into the imaging spectrometer via a narrow entrance slit. The illumination source in this instrument was a white light lamp or a broadband light-emitting diode (LED) in the NIR spectral range, and the detector was a CCD camera. In a single CCD image (Figure 2A, bottom-right inset), the reflectance spectra of several hundred independent locations in one line that spans the PC were gathered at one time. To construct a 2D PWV image, a scanning stage translated the PC across the illumination line in small spatial increments.

The first generation of scanning PC imaging instruments (BIND Scanner) was developed into a commercially available product and utilized in many life science research applications [38,39,40,41,43,50,80,82,83]. For example, it has been reported in [83] that assessing combined enhanced fluorescence and label-free biomolecular detection on the same PC surface. The sensitivity of the PC biosensor has been examined in detail in [80]. The PWS image shown in Figure 2B illustrates the detection of a microarray of Protein A printed on the biosensor surface to form the letters ‘NSG’ (Nano Sensors Group, University of Illinois at Urbana-Champaign) [50]. Cytotoxicity screening of Bangladeshi medicinal plant extracts has been performed with pancreatic cancer cells (Panc-1) using the BIND Scanner. As shown in Figure 2C, the untreated control group and two representative plant extracts, Petunia punctata Paxton (*P. punctate*) and Anisoptera glabra Kurz (*A. glabra*), demonstrate different cellular activities (apoptosis and proliferation) on the biosensor surfaces [41]. The imaging instrument was sufficient for observing large populations of cells with ~9 µm spatial resolution, so that cells with large surface attachment footprints could be observed, although the system lacked sufficient resolution for observing intra-cell attachment dynamics.

### 3.2. Instrument 2—Transmission Acquisition Mode with Upright Microscopy and Laser Source

To improve spatial resolution, an upright microscope (Olympus BX-51WI) was integrated into the PC imaging system in 2009 [76] and the resulting system was named the “Photonic Crystal Enhanced Microscope” [44]. Instead of measuring reflection efficiency as a function of wavelength from the bottom of the PC surface, the second generation PCEM measured transmission efficiency as a function of incident angle, using a fixed illumination wavelength from a beam-expanded laser (Instrument 2, Figure 3A). This instrument was designed as a wide-field imaging system with collimated angle-tunable laser illumination, which allowed imaging of a PC surface using the same illumination source and imaging optics for both enhanced fluorescence (EF) and label-free (LF) modalities. As shown in Figure 3A, the light beam generated from a HeNe laser passes through a half-wave plate (for polarization control), a variable neutral density filter, a rotating diffuser (to reduce speckle and fringes), a beam expander, an aperture, and a motorized angle-tunable mirror before passing through the PC (which is positioned beneath the microscope objective lens). The gimbal-mounted motorized mirror sits on top of a motorized linear stage in order to maintain a constant illumination area on the PC device (as the mirror rotates) and provide selective light coupling to the PC. Using this approach, high spatial-resolution and high sensitivity LF and EF images (Figure 3B) can be accurately registered with each other since a common beam-path is shared for both imaging modes [76]. An electron-multiplying (EM) CCD camera was used to acquire high resolution and large-area images, and thus enable high-throughput analysis. Moreover, images can be simultaneously acquired with other imaging techniques available on the EF/LF microscope, including reflected brightfield (BF) and differential interference contrast (DIC) images that can be overlaid with EF and LF images.

This transmission-based PC imaging modality that was capable of simultaneous label-free and enhanced fluorescence imaging (EF/LF) was further developed and utilized in several follow-up publications [20,41,42,44,76,81,84]. One of the main applications envisioned for the instrument was for performing DNA and protein microarray analysis, in which the label-free image of immobilized capture spots could be used to verify correct microarray printing and uniform spot density, while the enhanced fluorescence imaging modality would be used after hybridization of the target molecules from a test sample that carries fluorescent tags. Optimization of the imaging spatial resolution was reported in [81]. Microplate, microfluidic channel, and spot-based affinity capture assays were also demonstrated with this detection platform [84]. Figure 3C shows an example of a label-free image acquired with a tunable resonant angle for a DNA microarray immobilized on the biosensor surface [37]. Figure 3D shows a line profile through a row (red line in Figure 3C) containing 4 blank spots followed by 12 probe spots. It can be clearly observed that areas where the probe DNA has been immobilized produce a measurable increase in the resonant angle.

### 3.3. Instrument 3—Reflection Acquisition Mode with Inverted Microscopy and LED Source

Recently, the PCEM instrumentation transitioned to its third generation, in which an inverted microscope (Carl Zeiss Axio Observer Z1) body was chosen as the base of the system (Instrument 3, Figure 4A,B) [45,48]. While the second generation PCEM was developed specifically for scanning PC surfaces in a dry state for the detection of surface-adsorbed biomolecule patterns (such as DNA microarrays), the third generation PCEM was designed for label-free detection of cells and real-time detection of binding events in which the PC surface is exposed to liquid. In order to avoid scattering and absorption or interference from cell bodies, microfluidic components, semi-opaque liquid media, or liquid-air meniscus, bottom illumination of the PC was adopted in a reflection mode. In this system, detection of resonant reflected wavelength shifts was adopted again as the sensing approach rather than sensing changes in the resonant angle for a fixed illumination wavelength. An LED was chosen as the light source to avoid the speckles in the acquired images that may be caused by a laser illumination source. To obtain higher illumination intensity from the LED light source, a cylindrical lens was added into the illumination light path to convert the incident light from a circular spot to a more concentrated linear beam [45].

Label-free imaging of surface-absorbed live cells (including cell attachment, chemotaxis, and apoptosis) [45] and nanoparticles [48] has been performed using the third generation PCEM. Fluorescence-labeled imaging is also enabled in this system, in which the PC can be excited by a laser illumination source that can couple with the resonant PC mode to obtain an electric field enhancement effect. This enhancement is capable of increasing fluorescence detection sensitivity (which has been validated previously [20,76,83,85,86,87,88,89,90,91,92,93,94,95]) and enabling estimation of the distance of fluorescence emitters from the PC surface [96]. The most recently adopted PC surface design and PCEM detection instrument configurations have already been described in detail in section 2.

## 4. Applications of PCEM

The PCEM imaging system can be applied to monitor kinetic changes in the spatial distribution of dielectric permittivity for surface-adsorbed materials. This section describes PCEM applications with several examples, such as label-free live cell imaging, nanoparticle and protein-protein binding detection, and intensity enhancement of fluorescent tags embedded within live cells.

### 4.1. PCEM for Label-Free Live Cell Imaging

Label-free live cell imaging involves a sensing transducer surface, which typically generates an electrical or optical signal when cells interact with it. Biosensors that measure intrinsic cellular properties (such as dielectric permittivity) can be used to determine the number of cells in contact with the transducer, or the distribution/redistribution of focal adhesion areas. Such transducers (e.g., PC biosensors) may be prepared with different surface chemistry coatings that either mimic the *in vivo* microenvironment within tissues or selectively capture specific cell populations through interaction with proteins expressed on their outer cellular membranes. Therefore, the PCEM-based label-free images of cell attachment can assist the study of cell-substrate interactions, including identifying, capturing, and quantifying cells expressing specific surface molecules (Figure 2C) [38,39,40,41,42,43,44,45,50].

**Figure 5:**
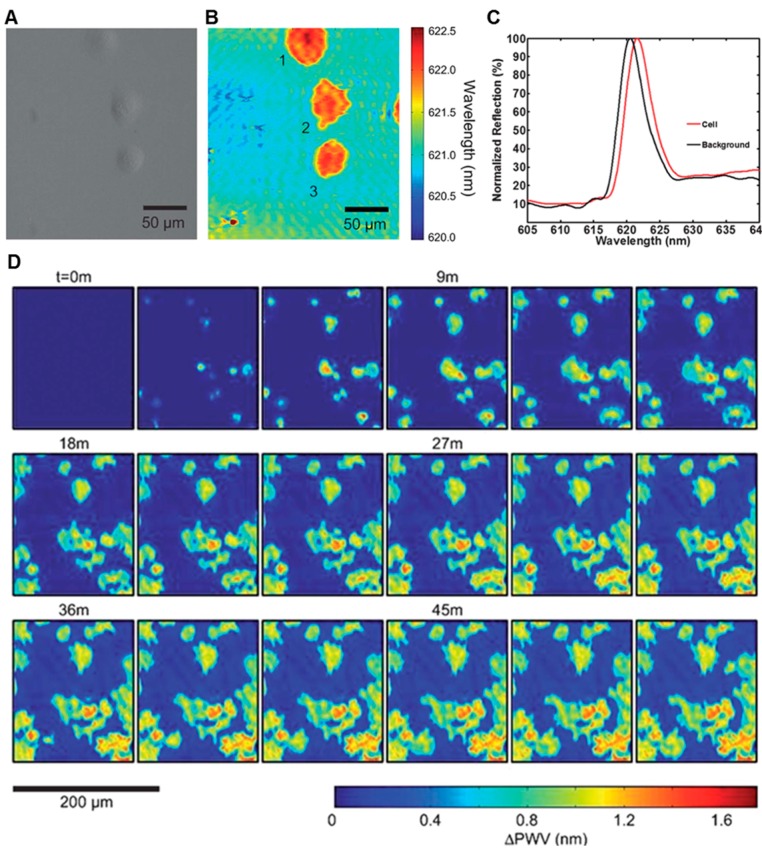
Wavelength-sensitive live cell image from instrument 3–PCEM. (**A**) Brightfield and (**B**) PWV images of Panc-1 cells attached to the PC surface. Lamellipodial extensions are visible, especially from cell 2, demonstrating the ability of PCEM to resolve regional differences in single-cell attachment; (**C**) Representative spectra (normalized) from background regions and regions with cellular attachment. Selected areas of the PWV image from beneath a cell show the PWS of a typical Panc-1 cell is ~1.0 nm; (**D**) Time-lapse PWS images of cellular attachment of dental stem cells (mHAT9a) (Reprinted in part with permission from [45], © 2013 RSC Publishing).

Recently, PCEM has been successfully demonstrated as a label-free live cell imaging approach to provide visualizations of each individual cell with subcellular details [45]. As shown in Figure 5A–C, Panc-1 cells were seeded onto a fibronectin-coated PC biosensor and allowed to incubate for 2 h before imaging. The non-uniform distribution of the PWS and the subcellular activity can be visualized clearly for each single cell. Figure 5B shows that the middle cell (No. 2) demonstrates higher PWS in regions near the boundary of lamellipodia formation (consistent with the creation of actin bundles). These darker shadings in the cell indicate regions of higher protein concentration, which may be attributed to higher modulation in the strength of cellular material attachment.

In addition, the kinetics of dynamic interaction between cellular materials and surface coating materials can be measured quantitatively using PCEM. As shown in Figure 5D, a sequence of movie frames demonstrates murine dental stem cells (mHAT9a) gradually attaching on the PC surface. Cells were seeded at 20,000 cells per mL on a fibronectin-coated PC biosensor surface. After three minutes, initial cell attachment appears as small, round regions, which is consistent with spheroid, trypsinized cells coming out of suspension and attaching to a surface. As time progresses, both the size of the cells and intensity of the PWS induced by them increases, indicating a higher localization of cellular material at the biosensor surface, which can be expected during cell spreading. Finally, once cells are sufficiently attached, cellular processes can be observed sensing the cells’ microenvironment in all directions. The outer irregular boundaries of the cells have a relatively low PWS (consistent with thin, exploratory filopodia) accompanied by a more heavily attached region slightly immediately adjacent in the cell interior (likely a result of actin bundle formation). Figure 5D illustrates distinct modulation distributions of the attachment strength for both individual cells and the whole cell group during different periods of the adhesion procedure.

### 4.2. PCEM for Imaging of Nanoparticle and Protein-Protein Binding

Because the PC surface structure restricts lateral propagation of light at the resonant wavelength, it is possible to create spatial maps of the resonant wavelength and the resonant damping that allow high spatial resolution imaging of small-size biomaterials distributed across the surface. Particles smaller than the pixel size (e.g., 600^2^ nm^2^ for our current PCEM) are very challenging to visualize and identify. However, it is possible to detect the presence of individual particles when the PWS induced by each particle is higher than the detection sensitivity limit of PCEM at each pixel location (the noise-induced PWS need to be considered as well). It is noteworthy that the PWV image for a particle is always within a diffraction-limited distance of up to five (or more) adjacent pixels and, hence, it is not expected to observe a PWS of only one pixel when a sub-micron nanoparticle attaches to the PC. As shown in Figure 4F, a PWV image is acquired for a 3 × 3 polystyrene particle array that is printed by thermal Dip-Pen Nanolithography (tDPN) [97,98] with heated atomic force microscopy (AFM) tips. Each particle has the dimension of ~540^2^ × 40 nm^3^ and ~5 µm gaps in between (Figure 4E). Figure 4G demonstrates two acquired spectra (one from a pixel at particle location, and one from background location) and each printed particle can cause ~0.5 nm PWS, which can be easily detected and visualized using the PCEM system. Not only dielectric nanoparticles (as optical scatters) but also metal nanoparticles (as optical absorbers) as small as ~100 nm can be detected via PIV-shift images using PCEM [48].

**Figure 6:**
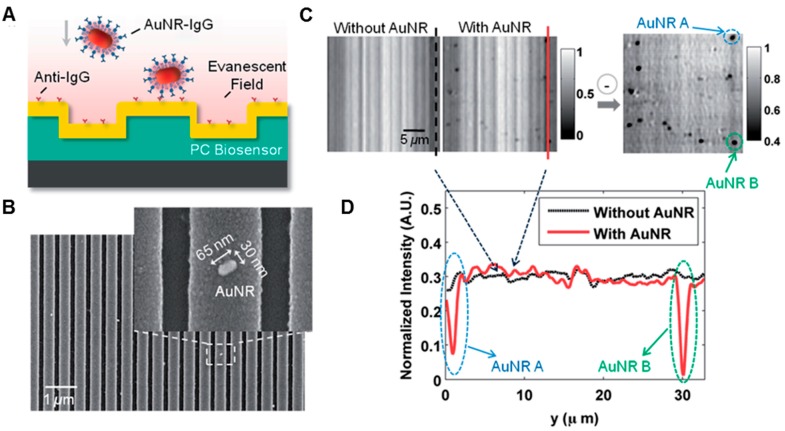
PCEM detection of protein-protein binding. (**A**) Schematic illustration of the PCEM detection of protein-protein binding on the PC biosensor surface; (**B**) SEM images of AuNR-IgG (AuNR conjugated with SH-PEG-IgG) attached to the PC biosensor surface. Inset: zoomed-in image for one AuNR; (**C**) PCEM-detected peak intensity value (PIV) images (in grayscale) and the PIV-shift image indicating AuNR-IgG attached on the PC surface; (**D**) Two representative cross-section lines of the normalized intensity images with/without two AuNRs-IgG on the PC surface (Reprinted in part with permission from [48], © 2014 RSC Publishing.).

Single nanoparticles allowing direct visualization in PCEM can be applied as biosensing tags to detect protein-protein binding for multiple events on a large sensor surface synchronously. This detection and imaging capability may be used in high-throughput screening during extended periods while avoiding photobleaching issues that are inherent for fluorescence dye tags. Furthermore, the resonance wavelength of nanoparticles can be conveniently tuned through the incident angle of the illumination light [44], the dimension of the PC biosensor [80], and the size or geometry of the nanoparticle [48,99,100]. An example is plotted in Figure 6 for PCEM detection of a target protein molecule (e.g., Rabbit Immunoglobulin G (IgG)) binding with the immobilized capture antibodies (e.g., anti-Rabbit IgG) using gold nanorods (AuNR) as tags [48]. The aspect ratio of the AuNR (dimension of ~65^2^ × 30 nm^3^) was tuned such that its localized surface plasmon resonance (LSPR) matched the resonant wavelength of the PC biosensor, and thus further improved the signal-to-noise ratio performance of the imaging system.

**Figure 7:**
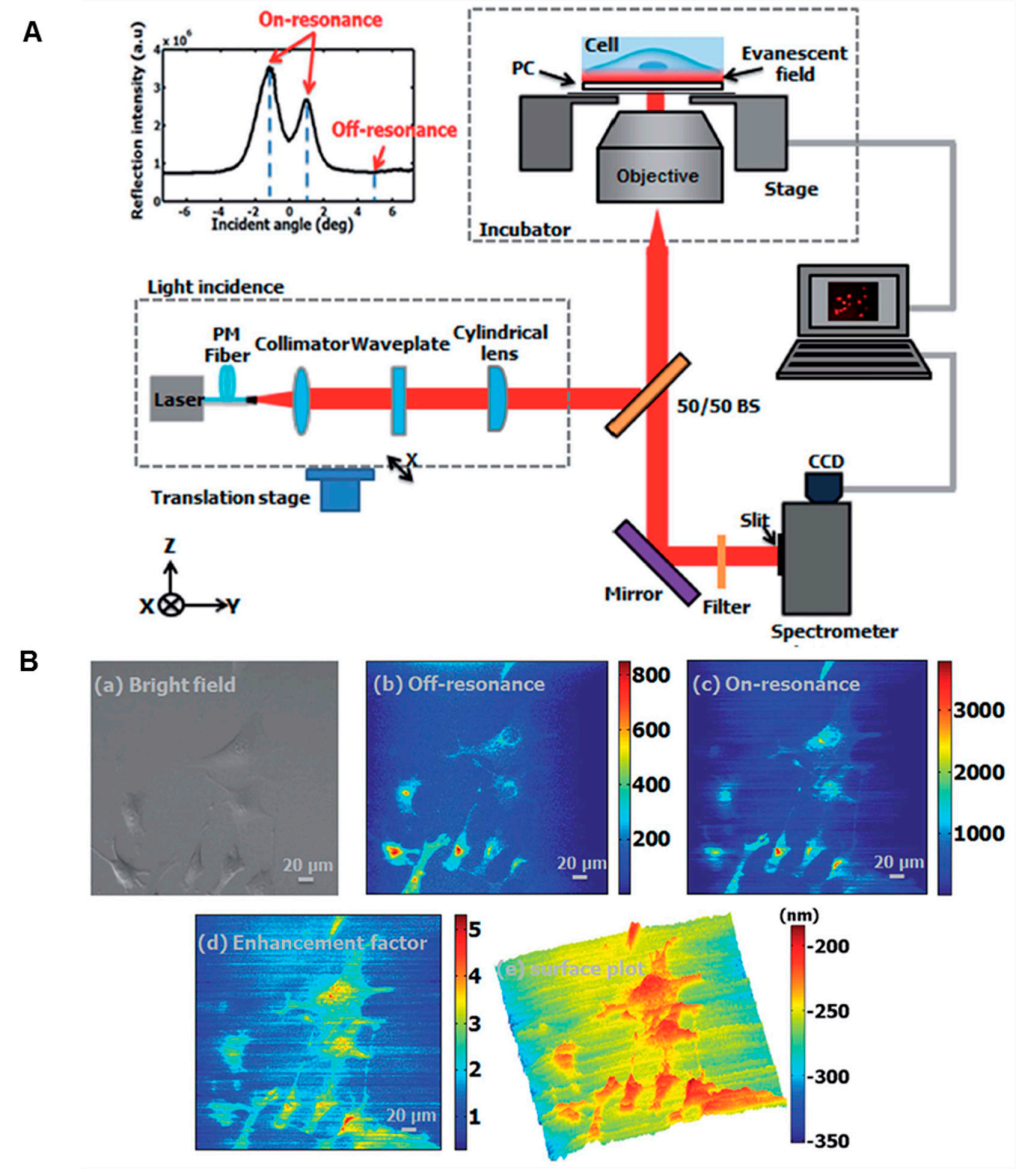
Photonic Crystal Enhanced Fluorescence (PCEF) portion on a PCEM imaging system. (**A**) Schematic of the PCEF portion on modern PCEM detection instrumentation. Inset (top left): angle reflection spectrum; (**B**) Brightfield and PCEF images of membrane dye-stained 3T3 fibroblast cells: (a) brightfield, (b) off-resonance PCEF, (c) on-resonance PCEF, (d) enhancement factor image, (e) 3D surface plot image of the enhancement factor (Reprinted in part with permission from [96], © 2014 RSC Publishing).

### 4.3. Combination of PCEM and PCEF for Label-Free/Fluorescence-Labeled Imaging *S*imultaneously

The PCEM is not limited to detection of optical scatters or absorbers, but is also capable of enhancing the emission and extraction from optical emitters (such as fluorescent dyes) in the evanescent field of the PC biosensor. Based on this principle, the label-free PCEM system can be slightly modified to include an additional illumination path from a laser that can excite fluorescent emitters. The ability to tune the illumination angle of the laser to match the resonant coupling condition of the PC substantially enhances the electric field intensity that is used to excite fluorophores, resulting in higher intensity fluorescence microscope images. Photonic Crystal Enhanced Fluorescence (PCEF) represents an additional imaging modality within the PCEM that enables rapid switching between label-free and fluorescence-labeled imaging modes (Figure 3A) [76,83]. Figure 3B demonstrates the enhanced fluorescence image and the label-free image of the same microarray spots printed with cyanine-5-tagged streptavidin (Cy5-SA) proteins. Figure 7A depicts the current optical setup for the PCEF portion of a combined imaging system. Illumination from a fiber-coupled semiconductor laser diode is collimated and passed through a half waveplate to produce a polarization perpendicular to the PC grating lines. Figure 7A inset (top left) plots an angle reflection spectrum of the PC surface when illuminated with a collimated semiconductor laser at 637 nm over a range of illumination angles. Maximum reflection intensity occurs at the on-resonance condition at an incident angle of ±1.14° from normal direction. The off-resonance condition refers to the laser illumination at an incidence angle of 5°. Figure 7B illustrates the corresponding enhanced fluorescence images for membrane dye-stained 3T3 fibroblast cells [96]. The combination of both modalities extends the PC-enhanced imaging system to be multi-functional and capable of imaging in numerous bio-applications.

## 5. Summary

Nanophotonic surfaces used in label-free biosensing and bioimaging are an attractive research area and have been involved in many biological applications, including disease diagnostics, drug discovery, and the fundamental study of molecular and cellular activity/function. Detection and imaging tools utilizing nanophotonic surfaces (such as PCEM) with high sensitivity, high detection throughput, and inexpensively manufactured sensors are demanding requirements for life science research and drug discovery applications. This paper reviewed the principles and applications along with the development history of PCEM, which utilizes a photonic crystal surface as an optical transducer to detect and visualize surface-absorbed biomaterials. PCEM achieves high sensitivity and high spatial-resolution due to the narrow spectra line width, restricted lateral propagation and evanescent field enhancement on the PC surface. The PC-enhanced imaging system can be applied to the quantitative and dynamic measurement of cell-substrate interactions, nanoparticle attachment, and protein-protein binding on the biosensor surface. PCEM can also be combined with PCEF to construct a versatile imaging system for tracking and visualizing different optical phenomena that occur within an individual sample. This novel imaging system opens new routes for the detection and visualization of surface-attached biomaterials and holds great potential to help uncover numerous underlying biological mechanisms.

## References

[1] Hessel A., Oliner A.A. (1965). A new theory of wood’s anomalies on optical gratings. Appl. Opt..

[2] Mashev L., Popov E. (1984). Diffraction efficiency anomalies of multicoated dielectric gratings. Opt. Commun..

[3] Popov E., Mashev L., Maystre D. (1986). Theoretical study of the anomalies of coated dielectric gratings. Opt. Acta.

[4] Yablonovitch E. (1987). Inhibited spontaneous emission in solid-state physics and electronics. Phys. Rev. Lett..

[5] John S. (1987). Strong localization of photons in certain disordered dielectric superlattices. Phys. Rev. Lett..

[6] Joannopoulos J.D., Villeneuve P.R., Fan S. (1997). Photonic crystals: Putting a new twist on light. Nature.

[7] Fan S.H., Joannopoulos J.D. (2002). Analysis of guided resonances in photonic crystal slabs. Phys. Rev. B.

[8] Joannopoulos J.D., Johnson S.G., Winn J.N., Meade R.D. (2008). Photonic Crystals: Molding the Flow of Light.

[9] Magnusson R., Wang S.S. (1992). New principle for optical filters. Appl. Phys. Lett..

[10] Kikuta H., Maegawa N., Mizutani A., Iwata K., Toyota H. (2001). Refractive index sensor with a guided-mode resonant grating filter. Proc. SPIE.

[11] Villa F., Regalado L.E., Ramos-Mendieta F., Gaspar-Armenta J., Lopez-Rios T. (2002). Photonic crystal sensor based on surface waves for thin-film characterization. Opt. Lett..

[12] Cunningham B.T., Li P., Schulz S., Lin B., Baird C., Gerstenmaier J., Genick C., Wang F., Fine E., Laing L. (2004). Label-free assays on the bind system. J. Biomol. Screen..

[13] Fang Y., Ferrie A.M., Fontaine N.H., Mauro J., Balakrishnan J. (2006). Resonant waveguide grating biosensor for living cell sensing. Biophys. J..

[14] Skivesen N., Tetu A., Kristensen M., Kjems J., Frandsen L.H., Borel P.I. (2007). Photonic-crystal waveguide biosensor. Opt. Expr..

[15] Konopsky V.N., Alieva E.V. (2007). Photonic crystal surface waves for optical biosensors. Anal. Chem..

[16] Nazirizadeh Y., Geyer U., Lemmer U., Gerken M. Spatially resolved optical characterization of photonic crystal slabs using direct evaluation of photonic modes. Proceedings of  the IEEE International Conference on Optical MEMs and Nanophotonics.

[17] Guo Y.B., Divin C., Myc A., Terry F.L., Baker J.R., Norris T.B., Ye J.Y. (2008). Sensitive molecular binding assay using a photonic crystal structure in total internal reflection. Opt. Expr..

[18] Fang Y., Frutos A.G., Verklereen R. (2008). Label-free cell-based assays for gpcr screening. Comb. Chem. High Throughput Screen..

[19] Konopsky V.N., Alieva E.V. (2009). Optical biosensors based on photonic crystal surface waves. Methods Mol. Biol..

[20] Cunningham B.T. (2010). Photonic crystal surfaces as a general purpose platform for label-free and fluorescent assays. JALA Charlottesv Va.

[21] El Beheiry M., Liu V., Fan S., Levi O. (2010). Sensitivity enhancement in photonic crystal slab biosensors. Opt. Expr..

[22] Nazirizadeh Y., Bog U., Sekula S., Mappes T., Lemmer U., Gerken M. (2010). Low-cost label-free biosensors using photonic crystals embedded between crossed polarizers. Opt. Expr..

[23] Jamois C., Li C., Gerelli E., Orobtchouk R., Benyattou T., Belarouci A., Chevolot Y., Monnier V., Souteyrand E., Serra P.A. (2011). New Concepts of Integrated Photonic Biosensors Based on Porous Silicon. Biosensors-Emerging Materials and Applications.

[24] Magnusson R., Wawro D., Zimmerman S., Ding Y. (2011). Resonant photonic biosensors with polarization-based multiparametric discrimination in each channel. Sensors.

[25] Nazirizadeh Y., Becker T., Reverey J., Selhuber-Unkel C., Rapoport D.H., Lemmer U., Gerken M. (2012). Photonic crystal slabs for surface contrast enhancement in microscopy of transparent objects. Opt. Expr..

[26] Pal S., Fauchet P.M., Miller B.L. (2012). 1-d and 2-d photonic crystals as optical methods for amplifying biomolecular recognition. Anal.Chem..

[27] Threm D., Nazirizadeh Y., Gerken M. (2012). Photonic crystal biosensors towards on-chip integration. J. Biophotonics.

[28] Carbonell J., Diaz-Rubio A., Torrent D., Cervera F., Kirleis M.A., Pique A., Sanchez-Dehesa J. (2012). Radial photonic crystal for detection of frequency and position of radiation sources. Sci. Rep..

[29] Grepstad J.O., Kaspar P., Solgaard O., Johansen I.R., Sudbo A.S. (2012). Photonic-crystal membranes for optical detection of single nano-particles, designed for biosensor application. Opt. Expr..

[30] Troia B., Paolicelli A., Leonardis F.D., Passaro V.M.N., Passaro V.M.N. (2013). Photonic crystals for optical sensing: A review. Advances in Photonic Crystals.

[31] Lin B., Qiu J., Gerstenmeier J., Li P., Pien H., Pepper J., Cunningham B. (2002). A label-free optical technique for detecting small molecule interactions. Biosens. Bioelectron..

[32] Chan L.L., Cunningham B.T., Li P.Y., Puff D. (2006). A self-referencing method for microplate label-free photonic-crystal biosensors. IEEE Sens. J..

[33] Chan L.L., Lidstone E.A., Finch K.E., Heeres J.T., Hergenrother P.J., Cunningham B.T. (2009). A method for identifying small molecule aggregators using photonic crystal biosensor microplates. J. Assoc. Lab. Autom..

[34] Ge C., Lu M., George S., Flood T.A., Wagner C., Zheng J., Pokhriyal A., Eden J.G., Hergenrother P.J., Cunningham B.T. (2013). External cavity laser biosensor. Lab Chip.

[35] Zhang M., Peh J., Hergenrother P.J., Cunningham B.T. (2014). Detection of protein-small molecule binding using a self-referencing external cavity laser biosensor. J. Am. Chem. Soc..

[36] Shafiee H., Lidstone E.A., Jahangir M., Inci F., Hanhauser E., Henrich T.J., Kuritzkes D.R., Cunningham B.T., Demirci U. (2014). Nanostructured optical photonic crystal biosensor for HIV viral load measurement. Sci. Rep..

[37] George S., Block I.D., Jones S.I., Mathias P.C., Chaudhery V., Vuttipittayamongkol P., Wu H.Y., Vodkin L.O., Cunningham B.T. (2010). Label-free prehybridization DNA microarray imaging using photonic crystals for quantitative spot quality analysis. Anal. Chem..

[38] Lin B., Li P., Cunningham B.T. (2006). A label-free biosensor-based cell attachment assay for characterization of cell surface molecules. Sens. Actuators B Chem..

[39] Chan L.L., Gosangari S.L., Watkin K.L., Cunningham B.T. (2007). A label-free photonic crystal biosensor imaging method for detection of cancer cell cytotoxicity and proliferation. Apoptosis.

[40] Chan L.L., Gosangari S.L., Watkin K.L., Cunningham B.T. (2008). Label-free imaging of cancer cells using photonic crystal biosensors and application to cytotoxicity screening of a natural compound library. Sens. Actuators B Chem..

[41] George S., Bhalerao S.V., Lidstone E.A., Ahmad I.S., Abbasi A., Cunningham B.T., Watkin K.L. (2010). Cytotoxicity screening of bangladeshi medicinal plant extracts on pancreatic cancer cells. Complement. Altern. Med..

[42] Shamah S.M., Cunningham B.T. (2011). Label-free cell-based assays using photonic crystal optical biosensors. Analyst.

[43] Chan L.L., George S., Ahmad I., Gosangari S.L., Abbasi A., Cunningham B.T., Watkin K.L. (2011). Cytotoxicity effects of amoorarohituka and chittagonga on breast and pancreatic cancer cells. Complement. Altern. Med..

[44] Lidstone E.A., Chaudhery V., Kohl A., Chan V., Wolf-Jensen T., Schook L.B., Bashir R., Cunningham B.T. (2011). Label-free imaging of cell attachment with photonic crystal enhanced microscopy. Analyst.

[45] Chen W.L., Long K.D., Lu M., Chaudhery V., Yu H., Choi J.S., Polans J., Zhuo Y., Harley B.A.C., Cunningham B.T. (2013). Photonic crystal enhanced microscopy for imaging of live cell adhesion. Analyst.

[46] Cunningham B.T., Qiu J., Li P., Pepper J., Hugh B. (2002). A plastic colorimetric resonant optical biosensor for multiparallel detection of label-free biochemical interactions. Sens. Actuators B Chem..

[47] Li P., Lin B., Gerstenmaier J., Cunningham B.T. (2004). A new method for label-free imaging of biomolecular interactions. Sens. Actuators B Chem..

[48] Zhuo Y., Hu H., Chen W.L., Lu M., Tian L.M., Yu H.J., Long K.D., Chow E., King W.P., Singamaneni S. (2014). Single nanoparticle detection using photonic crystal enhanced microscopy. Analyst.

[49] Cunningham B., Qiu J., Li P., Lin B. (2002). Enhancing the surface sensitivity of colorimetric resonant optical biosensors. Sens. Actuators B Chem..

[50] Cunningham B.T., Laing L. (2006). Microplate-based, label-free detection of biomolecular interactions: Applications in proteomics. Expert Rev. Proteom..

[51] Cunningham B.T., Li P., Lin B., Pepper J. (2002). Colorimetric resonant reflection as a direct biochemical assay technique. Sens. Actuators B Chem..

[52] Yeh P., Yariv A., Cho A.Y. (1978). Optical surface waves in periodic layered media. Appl. Phys. Lett..

[53] Meade R.D., Brommer K.D., Rappe A.M., Joannopoulos J.D. (1991). Electromagnetic bloch waves at the surface of a photonic crystal. Phys. Rev. B.

[54] Robertson W.M., May M.S. (1999). Surface electromagnetic wave excitation on one-dimensional photonic band gap arrays. Appl. Phys. Lett..

[55] Shinn M., Robertson W.M. (2005). Surface plasmon-like sensor based on surface electromagnetic waves in a photonic band gap material. Sens. Actuators B Chem..

[56] Descrovi E., Frascella F., Sciacca B., Geobaldo F., Dominici L., Michelotti F. (2007). Coupling of surface waves in highly defined one-dimensional porous silicon photonic crystals for gas sensing applications. Appl. Phys. Lett..

[57] Sfez T., Descrovi E., Dominici L., Nakagawa W., Michelotti F., Giorgis F., Herzig H.P. (2008). Near-field analysis of surface electromagnetic waves in the bandgap region of a polymeric grating written on a one-dimensional photonic crystal. Appl. Phys. Lett..

[58] Sinibaldi A., Danz N., Descrovi E., Munzertb P., Schulz U., Sonntag F., Dominici L., Michelotti F. (2012). Direct comparison of the performance of bloch surface wave and surface plasmon polariton sensors. Sens. Actuators B Chem..

[59] Li Y., Yang T., Pang Z., Du G., Song S., Han S. (2014). Phase-sensitive bloch surface wave sensor based on variable angle spectroscopic ellipsometry. Opt. Expr..

[60] Fan S., Villeneuve P.R., Joannopoulos J.D., Schubert E.F. (1997). High extraction efficiency of spontaneous emission from slabs of photonic crystals. Phys. Rev. Lett..

[61] Kanskar M., Paddon P., Pacradouni V., Morin R., Busch A., Young J.F., Johnson S.R., MacKenzie J., Tiedje T. (1997). Observation of leaky slab modes in an air-bridged semiconductor waveguide with a two-dimensional photonic lattice. Appl. Phys. Lett..

[62] Villeneuve P.R., Fan S., Johnson S.G., Joannopoulos J.D. (1998). Three-dimensional photon confinement in photonic crystals of low-dimensional periodicity. IEEE Proc. Optoelectron..

[63] Johnson S.G., Fan S., Villeneuve P.R., Joannopoulos J.D., Kolodziejski L.A. (1999). Guided modes in photonic crystal slabs. Phys. Rev. B.

[64] Painter O., Vuckovic J., Scherer A. (1999). Defect modes of a two-dimensional photonic crystal in an optically thin dielectric slab. J. Opt. Soc. Am. B.

[65] Boroditsky M., Vrijen R., Krauss T.F., Coccioli R., Bhat R., Yablonovitch E. (1999). Spontaneous emission extraction and purcell enhancement from thin-film 2-d photonic crystals. Lightwave Technol..

[66] Astratov V.N., Culshaw I.S., Stevenson R.M., Whittaker D.M., Skolnick M.S., Krauss T.F., de la Rue R.M. (1999). Resonant coupling of near-infrared radiation to photonic band structure waveguides. Lightwave Technol..

[67] Baba T., Fukaya N., Yonekura J. (1999). Observation of light propagation in photonic crystal optical waveguides with bends. Electron. Lett..

[68] Paddon P., Young J.F. (2000). Two-dimensional vector-coupled-mode theory for textured planar waveguides. Phys. Rev. B.

[69] Pacradouni V., Mandeville W.J., Cowan A.R., Paddon P., Young J.F., Johnson S.R. (2000). Photonic band structure of dielectric membranes periodically textured in two dimensions. Phys. Rev. B.

[70] Kuchinsky S., Allan D.C., Borrelli N.F., Cotteverte J.C. (2000). 3D localization in a channel waveguide in a photonic crystal with 2d periodicity. Opt. Commun..

[71] Lin S.Y., Chow E., Johnson S.G., Joannopoulos J.D. (2000). Demonstration of highly efficient waveguiding in a photonic crystal slab at the 1.5-um wavelength. Opt. Lett..

[72] Benisty H., Labilloy D., Weisbuch C., Smith C.J.M., Krauss T.F., Cassagne D., Beraud A., Jouanin C. (2000). Radiation losses of waveguide-based two-dimensional photonic crystals: Positive role of the substrate. Appl. Phys. Lett..

[73] Chutinan A., Noda S. (2000). Waveguides and waveguide bends in two-dimensional photonic crystal slabs. Phys. Rev. B.

[74] Cowan A.R., Paddon P., Pacradouni V., Young J.F. (2001). Resonant scattering and mode coupling in two-dimensional textured planar waveguides. J. Opt. Soc. Am. A.

[75] Vahala K. (2004). Optical Microcavities (Advanced Series in Applied Physics).

[76] Block I.D., Mathias P.C., Ganesh N., Jones I.D., Dorvel B.R., Chaudhery V., Vodkin L., Bashir R., Cunningham B.T. (2009). A detection instrument for enhanced fluorescence and label-free imaging on photonic crystal surfaces. Opt. Expr..

[77] Schulz S.C. (2008). Web based photonic crystal biosensors for drug discovery & diagnostics. Vac. Coat..

[78] Krebs F.C. (2009). Polymer solar cell modules prepared using roll-to-roll methods: Knife-over-edge coating, slot-die coating and screen printing. Sol. Energy Mater. Sol. Cells.

[79] Ge C., Lu M., Jian X., Tan Y.F., Cunningham B.T. (2010). Large-area organic distributed feedback laser fabricated by nanoreplica molding and horizontal dipping. Opt. Expr..

[80] Block I.D., Ganesh N., Lu M., Cunningham B.T. (2008). A sensitivity model for predicting photonic crystal biosensor performance. IEEE Sens. J..

[81] Block I.D., Mathias P.C., Jones S.I., Vodkin L.O., Cunningham B.T. (2009). Optimizing the spatial resolution of photonic crystal label-free imaging. Appl. Opt..

[82] Choi C.J., Cunningham B.T. (2006). Single-step fabrication and characterization of photonic crystal biosensors with polymer microfluidic channels. Lab Chip.

[83] Mathias P.C., Ganesh N., Chan L.L., Cunningham B.T. (2007). Combined enhanced fluorescence and label-free biomolecular detection with a photonic crystal surface. Appl. Opt..

[84] Choi C.J., Belobraydich A.R., Chan L.L., Mathias P.C., Cunningham B.T. (2010). Comparison of label-free biosensing in microplate, microfluidic, and spot-based affinity capture assays. Anal. Biochem..

[85] Ganesh N., Zhang W., Mathias P.C., Chow E., Soares J.A., Malyarchuk V., Smith A.D., Cunningham B.T. (2007). Enhanced fluorescence emission from quantum dots on a photonic crystal surface. Nat. Nanotechnol..

[86] Ganesh N., Block I.D., Mathias P.C., Zhang W., Chow E., Malyarchuk V., Cunningham B.T. (2008). Leaky-mode assisted fluorescence extraction: Application to fluorescence enhancement biosensors. Opt. Expr..

[87] Ganesh N., Mathias P.C., Zhang W., Cunningham B.T. (2008). Distance dependence of fluorescence enhancement from photonic crystal surfaces. J. Appl. Phys..

[88] Pokhriyal A., Lu M., Huang C.S., Schulz S., Cunningham B.T. (2010). Multicolor fluorescence enhancement from a photonics crystal surface. Appl. Phys. Lett..

[89] Pokhriyal A., Lu M., Chaudhery V., Huang C.S., Schulz S., Cunningham B.T. (2010). Photonic crystal enhanced fluorescence using a quartz substrate to reduce limits of detection. Opt. Expr..

[90] Mathias P.C., Ganesh N., Zhang W., Cunningham B.T. (2008). Graded wavelength one-dimensional photonic crystal reveals spectral characteristics of enhanced fluorescence. J. Appl. Phys.

[91] Mathias P.C., Wu H.Y., Cunningham B.T. (2009). Employing two distinct photonic crystal resonances to improve fluorescence enhancement. Appl. Phys. Lett..

[92] Wu H.Y., Zhang W., Mathias P.C., Cunningham B.T. (2010). Magnification of photonic crystal fluorescence enhancement via tm resonance excitation and te resonance extraction on a dielectric nanorod surface. Nanotechnology.

[93] Chaudhery V., Lu M., Pokhriyal A., Schulz S.C., Cunningham B.T. (2012). Angle-scanning photonic crystal enhanced fluorescence microscopy. IEEE Sens. J..

[94] George S., Chaudhery V., Lu M., Takagi M., Amro N., Pokhriyal A., Tan Y.F., Ferreira P., Cunningham B.T. (2013). Sensitive detection of protein and mirna cancer biomarkers using silicon-based photonic crystals and a resonance coupling laser scanning platform. Lab Chip.

[95] Pokhriyal A., Lu M., Ge C., Cunningham B.T. (2014). Coupled external cavity photonic crystal enhanced fluorescence. J. Biophotonics.

[96] Chen W.L., Long K.D., Yu H.J., Tan Y.F., Choi J.S., Harley B.A., Cunningham B.T. (2014). Enhanced live cell imaging via photonic crystal enhanced fluorescence microscopy. Analyst.

[97] Hu H., Mohseni P.K., Pan L., Li X., Somnath S., Felts J.R., Shannon M.A., King W.P. (2013). Fabrication of arbitrarily-shaped silicon and silicon oxide nanostructures using tip-based nanofabrication. J. Vac. Sci. Technol. B.

[98] King W.P., Bhatia B., Felts J.R., Kim H.J., Kwon B., Lee B., Somnath S., Rosenberger M. (2013). Heated atomic force microscope cantilevers and their applications. Annu. Rev. Heat Transf..

[99] Tian L., Chen E., Gandra N., Abbas A., Singamaneni S. (2012). Gold nanorods as plasmonic nanotransducers: Distance-dependent refractive index sensitivity. Langmuir.

[100] Tian L., Morrissey J.J., Kattumenu R., Gandra N., Kharasch E.D., Singamaneni S. (2012). Bioplasmonic paper as a platform for detection of kidney cancer biomarkers. Anal. Chem..

